# Complete Genome Sequence of *Methanobrevibacter smithii* Strain KB11, Isolated from a Korean Fecal Sample

**DOI:** 10.1128/genomeA.00038-18

**Published:** 2018-02-15

**Authors:** Byoung-Chan Kim, Haeyoung Jeong

**Affiliations:** a Metabolic Regulation Research Center, Korea Research Institute of Bioscience and Biotechnology (KRIBB), Daejeon, Republic of Korea; b Infectious Disease Research Center, Korea Research Institute of Bioscience and Biotechnology (KRIBB), Daejeon, Republic of Korea; c Department of Bioprocess Engineering, KRIBB School of Biotechnology, Korea University of Science and Technology (UST), Daejeon, Republic of Korea; d Department of Biosystems and Bioengineering, KRIBB School of Biotechnology, Korea University of Science and Technology (UST), Daejeon, Republic of Korea

## Abstract

The archaeon *Methanobrevibacter smithii* is a major colonizer of the human gut. *Methanobrevibacter smithii* strain KB11 was newly isolated from a Korean fecal sample. Here, we present the complete genome sequence of strain KB11 and a brief comparison with that of *M. smithii* type strain ATCC 35061^T^.

## GENOME ANNOUNCEMENT

*Methanobrevibacter* is a fastidious archaeal microorganism and obligate anaerobe. It can be cultivated in a pressurized anaerobic atmosphere of 80% hydrogen and 20% carbon dioxide (1, 2). Among *Methanobrevibacter* species, *M. smithii* is a near-ubiquitous resident of the human intestinal microbiome (3). The prevalence of methanogens in humans has been reported to be dependent on the age of the human host; populations increase gradually throughout childhood, reaching a maximum abundance level in adults (3, 4). *Methanobrevibacter smithii* may impact human health (5, 6), but its beneficial effect is still questionable. In the course of an investigation into the diversity of methanogens in the human gut, feces were collected with an anaerobic sampling system (7) and were maintained under strict anaerobic conditions, as previously described (8). A methanogenic strain, KB11, was purified from the feces of a healthy 43-year-old Korean male. This is the first human gut methanogen isolated from South Korea.

The genomic DNA of KB11 was extracted as previously described (2). Complete genome sequencing was carried out using Illumina MiSeq and PacBio RS II platforms, as recommended by the manufacturers, at ChunLab, Inc. (Seoul, Republic of Korea). A hybrid assembly was performed using SPAdes v3.9.0 (http://cab.spbu.ru/software/spades/) from a total of 1.98 Gb MiSeq reads (average length, 192.6 bp) and 850 Mb of PacBio RS II filtered subreads (mean length, 8,534 bp; *N*_50_, 11,609 bp), generated by HGAP 2.0. The largest contig (1.81-Mb long) was cut into pieces and joined using the CLC Genomics Workbench v9.5.1 (Qiagen Bioinformatics, Aarhus, Denmark), allowing the finalized sequence to begin with the *dnaA* gene. Self-corrected PacBio long reads were used for fragment joining and confirmation of the circular structure of the chromosomal contig. Residual errors were corrected by three successive rounds of MiSeq read mapping and consensus sequence extraction. Genome sequences were annotated using the NCBI Prokaryotic Genome Annotation Pipeline (https://www.ncbi.nlm.nih.gov/genome/annotation_prok/) and the Rapid Annotations using Subsystems Technology (RAST) server (9). To the best of our knowledge, this is the first reported publicly available complete genome sequence of *Methanobrevibacter smithii* isolated from a human gut (*M. smithii* ATCC 35061^T^ was isolated from a sewage digester [10]). The KB11 genome has a 1,805,545-bp chromosome (31.2% G+C), which is 47,615 bp smaller than that of ATCC 35061^T^. Despite the overall similarity, we found an inversion (699958 to 1484782) in the ATCC 35061^T^ chromosome that led to C-term truncation in the DNA helicase UvrD (Msm_0731). It is also noteworthy that an adhesin-like protein (Msm_0173, 2,879 amino acids [aa]) in ATCC 35061^T^ has 17 DUF11 domains (pfam01345), while the KB11 homolog is disrupted with a frameshift (BK798_03360). A KB11-specific region (500216 to 523396) was also found to accommodate genes encoding hypothetical proteins, a type I restriction-modification system, and transposases.

This additional genome information may be used to improve our understanding of the genetic diversity of human gut-associated *M. smithii* within individuals. This knowledge will enhance studies concerning the archaeal gut microbiota and human physiology, especially with respect to human health and disease.

### Accession number(s).

The complete genome sequences of *Methanobrevibacter smithii* KB11 have been deposited in DDBJ/ENA/GenBank under the accession number CP017803.
